# Immunoglobulin E (IgE)-mediated food allergy

**DOI:** 10.1186/s13223-024-00930-7

**Published:** 2024-12-30

**Authors:** Philippe Bégin, Susan Waserman, Jennifer L. P. Protudjer, Samira Jeimy, Wade Watson

**Affiliations:** 1 Division of Clinical Immunology and Allergy, Department of Medicine, Université de Montréal, Montréal, Québec Canada; 2 Division of Clinical Immunology and Allergy, Department of Medicine, McMaster University, Hamilton, ON Canada; 3 Department of Pediatrics and Child Health, College of Medicine, Rady Faculty of Health Sciences, University of Manitoba, Winnipeg, MB Canada; 4 Children’s Hospital Research Institute of Manitoba, Winnipeg, MB Canada; 5 Division of Clinical Immunology and Allergy, Department of Medicine, Western University, London, ON Canada; 6 Division of Allergy, Department of Pediatrics, Dalhousie University, IWK Health Centre, Halifax, NS Canada

## Abstract

Food allergy is defined as an adverse immunologic response to a food. Immunoglobulin E (IgE)-mediated reactions to foods are associated with a broad range of signs and symptoms that may involve any of the following body systems: the skin, gastrointestinal tract, respiratory tract, and cardiovascular system. IgE-mediated food allergy is a leading cause of anaphylaxis. Therefore, timely and appropriate diagnosis and treatment are imperative. A diagnosis of food allergy entails a careful history and diagnostic tests, which may include skin prick tests, serum-specific IgE, and oral food challenge. The goal of food allergy care is to empower patients and caregivers to manage the risk of food-allergic reactions, reduce food allergy-related anxiety, and achieve a sense of control over their condition. This can be achieved in different ways for different patients and across different life stages. This article provides an overview of the epidemiology, pathophysiology, diagnosis, and management of IgE-mediated food allergy.

## Key take-home messages

- Food allergy is defined as an adverse immunologic response to a food protein.
- Diagnosis of food allergy requires a detailed history and diagnostic tests, such as SPT and/or food-specific serum IgE measurement, and in some cases, OFCs.
- Management of food allergy involves avoidance of the culprit food(s) and self-injectable epinephrine for anaphylaxis.
- For patients with systemic symptoms, the treatment of choice is epinephrine administered by intramuscular injection into the lateral thigh.
- OIT is indicated as an option for food allergy management, which can be disease-modifying when performed early in the first years of life *(see OIT article in this supplement).* However, OIT is not readily accessible in many regions.

## Introduction

Immunoglobulin E (IgE)-mediated food allergy is a leading cause of anaphylaxis-related emergency department visits and is the most common cause of anaphylaxis in children [1–6] (see *Anaphylaxis* article in this supplement). In 2015, a survey of over 5700 Canadian households (15,022 individuals) estimated the prevalence of self-reported food allergy in Canada to be 7.5% (Table 1) [7]. Although any food can be an allergen, Health Canada has identified 11 priority “allergens” that food manufacturers are required to list on food labels. These include milk, egg, peanut, tree nuts, fish, shellfish (crustaceans and molluscs), wheat and triticale, sesame, soy, mustard, and sulphites [8]. Although a preservative and not technically a food allergen, sulphites have been added to this list since they can cause adverse reactions through non-immune mechanisms. “Hidden” food allergens, such as pea lupin protein, have also been associated with allergic reactions [9, 10] and are increasingly used as meat and gluten substitutes, respectively. Peas and lupin are genetically related to peanuts as they are part of the legume family, and some individuals with peanut allergy can also react to these allergens [9, 11, 12].

**Table 1:** Prevalence (self-reported, unadjusted) estimates for probable* food allergy in Canada [7]

	Prevalence (%)	
Food allergen	Children	Adults
Peanut	2.2	0.6
Tree nuts	1.5	1.0
Fish	0.9	0.5
Shellfish	0.8	1.6
Sesame	0.1	0.2
Milk	0.2	0.2
Egg	1.0	0.5
Wheat	0.2	0.2
Soy	0.1	0.1

Types of food allergies listed are not mutually exclusive

^*^Probable was defined as a convincing history and/or a physician diagnosis of IgE-mediated food allergy

Despite the increase in emergency department visits and hospital admissions, case fatalities due to food allergy have declined over the last 20 years, due to increased awareness and recognition of food allergy, and improved management of severe reactions [13, 14]. In the United States, approximately 150–200 deaths per year are attributed to food allergy [15]. A review of the 92 anaphylaxis-related deaths in Ontario, Canada, between 1986 and 2011 attributed 48% of these deaths to food allergy [14]. The investigators also observed a decline in the number of food-related fatalities over the 25-year period.

Accurate diagnosis and appropriate management of IgE-mediated food allergy are critical since, in some patients, accidental exposure to minute quantities of the culprit food may result in anaphylaxis (see *Anaphylaxis* article in this supplement), whereas others will tolerate the food despite positive tests [16]. This article reviews the definition, pathophysiology, diagnosis, and management of IgE-mediated food allergy.

### Definition

The term “food allergy” is used to describe an adverse immunologic response to a food. It is important to distinguish food allergy from other non-immune-mediated adverse reactions to foods, particularly since more than 20% of adults and children alter their diets due to perceived food allergy [17]. Adverse reactions to food that are not classified as food allergy may be secondary to metabolic disorders (e.g., lactose intolerance), reactions to toxic contaminants (e.g., bacteria in decomposing scombroid fish will convert histidine, an amino acid, to histamine), pharmacologically active food components (e.g., caffeine in coffee causing jitteriness, tyramine in aged cheeses triggering migraine), and conditions where symptoms are triggered by non-specific foods (e.g., irritable bowel disease, non-specific mast cell degranulation).

Food allergies are broadly categorized into those mediated by food-specific IgE antibodies and those resulting from other immune mechanisms initiated by food-specific T cells (non-IgE mediated or cell-mediated allergies) [17, 18]. IgE-mediated allergic responses are the most widely recognized form of food allergy and are characterized by the rapid onset of symptoms after ingestion of the culprit food. For a review of non-IgE-mediated food allergy, please see the article entitled, *Non-IgE-mediated Food Allergy,* in this supplement.

### Pathophysiology

With the exception of a carbohydrate known as galactose-alpha-1,3-galactose (also known as alpha-gal), it is the food protein, not the fat or carbohydrate content, that leads to sensitization and allergy [16–18]. The allergenic segments or epitopes of these proteins tend to be small [10–70 kilodaltons (kDa)], water-soluble glycoproteins that have varying degrees of resistance to denaturation by heat or acid. Some food allergen epitopes can remain intact after processing, storage, cooking, and digestion [16–18].

Food sensitization is the result of a previous exposure to the allergenic food proteins that leads to the production of food-specific IgE antibodies. In many cases, food sensitization is thought to occur through eczematous skin during infancy [19]. IgE antibodies are bound to basophils in blood and mast cells in tissues. Sensitization to a food can also result from cross-reactivity [20]. Cross-reactivity can occur when previous allergen-specific IgE antibodies recognize new food antigens that share structural similarities to the food or environmental allergen that had originally led to their production (see discussion of pollens and cross-reacting foods in next section). Subsequent exposure to the allergen can cause allergen-specific surface-bound IgE to aggregate and trigger intracellular signals, leading to mast cell degranulation and the release of histamine and other inflammatory mediators, resulting in clinical symptoms.

Clinical manifestations of IgE-mediated allergies depend on the route of exposure to the allergen. Following the ingestion of food, reactions can occur locally in the mouth and gut, or in distant organs, following the systemic absorption of the allergen(s). Direct contact with skin or eyes can result in local hives or swelling. Inhalation of aerosolized allergen from cooking (e.g. fish and shellfish) or flour dust can trigger asthma symptoms. However, the smell of the food alone does not cause an allergic reaction.

It is important to note that food sensitization, i.e., positive allergy testing, does not necessarily mean clinical reactivity [21]. Mast cell degranulation depends on various factors, including the concentration of allergen absorbed, its systemic neutralization by allergen-specific immunoglobulin G (IgG), affinity of the implicated IgE or mast cell intrinsic reactivity. In contrast, certain conditions, known as co-factors, can lead to more severe reactions to a given amount of allergen. Examples of co-factors include infection, exercise, alcohol, nonsteroidal anti-inflammatory drugs (NSAIDs) and other medications, stress, menstruation and sleep deprivation [17, 18, 22]. Some sensitized individuals will only experience a reaction when the allergen is eaten with one of these co-factors present, such as in food-associated exercise-induced anaphylaxis [23]. Some of these reactions may be unusually delayed if the co-factor occurs up to 4 h after ingestion [24].

Alpha-gal allergy is the only example of an IgE-mediated food allergy to a carbohydrate associated with anaphylaxis (see *Anaphylaxis* article in this supplement). It presents as a delayed allergic reaction to mammalian meat (e.g., beef, pork), which occurs approximately 3–6 h after ingestion [25]. Studies report that tick bites are the main cause of sensitization, since ticks inject alpha-gal through their saliva [25]. Although alpha-gal allergy is typically transient (lasting 3–5 years), it is an important consideration, particularly for those who require mammalian heart valves or some forms of chemotherapy (e.g., cetuximab) that contain alpha-gal [26, 27].

### Clinical manifestations

**Fig. 1:**
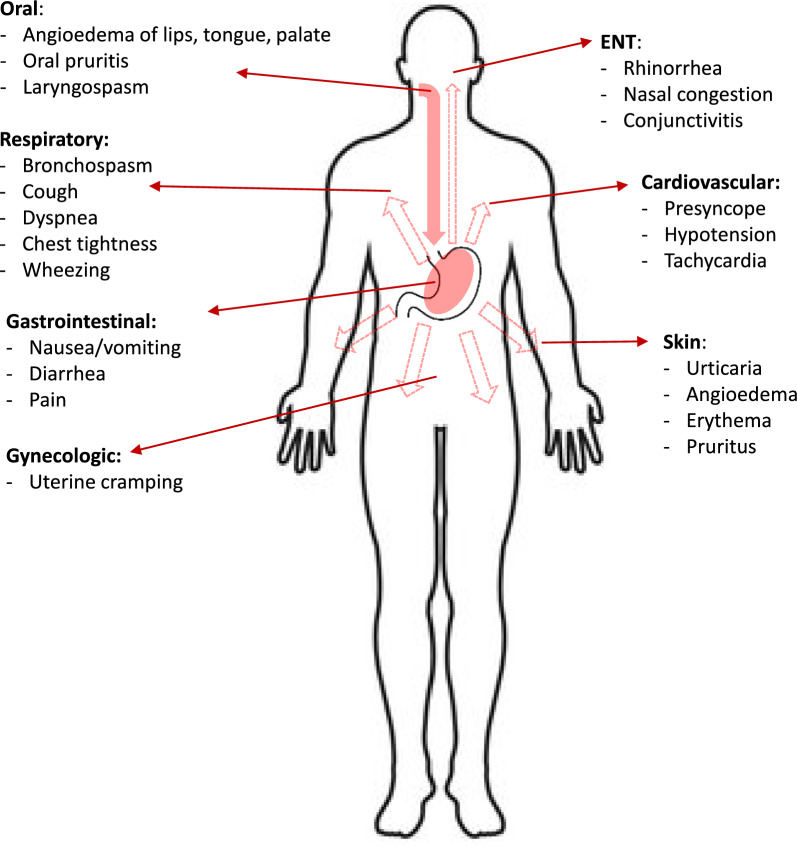
Signs and symptoms of IgE-mediated food allergy. ENT: ear, nose, throat

IgE-mediated food reactions are associated with a broad range of signs and symptoms that may involve any of the following body systems: the skin, GI tract, respiratory tract, and cardiovascular system (Fig. 1). Skin reactions, including urticaria, angioedema, and erythema, are the most common clinical manifestations of IgE-mediated food allergy. Common respiratory symptoms include laryngeal edema, rhinorrhea, and bronchospasm. Except in rare cases (e.g. baker’s asthma), IgE-mediated food allergy is not believed to play a role in chronic respiratory symptoms. GI-related signs and symptoms of food allergy include nausea, vomiting, abdominal pain, and diarrhea. Symptoms of IgE-mediated food allergy can appear within minutes to 2 h after food exposure, except in the case of alpha-gal allergy or co-factor dependent food allergies, where they can occur later.

The severity of a food allergic reaction can vary from person to person, and even from reaction to reaction in the same person. While the nature of symptoms in an individual tends to be similar from one reaction to another [28], their severity will typically follow a dose-dependent response, depending on the amount of food ingested and the presence of co-factors.

Allergy to heat-labile proteins will generally result in localized symptoms such as tingling and itching of the mouth and pharynx, which is characteristic of pollen-food allergy syndrome (PFAS), also known as oral allergy syndrome. In PFAS, symptoms are typically triggered by consumption of certain fresh fruits, vegetables, legumes, nuts and spices in pollen-allergic individuals (Table 2) [16, 29, 30]. The allergens are generally tolerated in their cooked forms, and, because the raw allergen is generally degraded by digestion, progression to systemic symptoms is rare, although it may occur in a small proportion (1–2%) of patients with this condition [29]. Hen’s egg and cow’s milk (CM) contain both heat-labile and heat-stable allergenic proteins. Therefore, depending on which specific protein the person is allergic to, they may or may not tolerate these foods if baked or cooked.

**Table 2:** Pollen-food allergy syndrome: pollens and cross-reacting foods [16, 30]

Pollen	Fresh fruit/vegetable/nuts/legumes/spices		
Birch	*Pitted fruits:* • Apple • Apricot • Cherry • Coconut • Nectarine • Peach • Pear • Plum *Other fruit:* • Kiwi • Tomato	*Vegetables:* • Carrot • Celery • Fennel • Parsley • Potato • Rutabaga/Swedish turnip	Legumes:* • Peanut • Soybean Nuts:* • Almond • Brazil nut • Hazelnut • Walnut
Ragweed	*Melons:* • Honeydew • Watermelon • Cantaloupe	*Other fruits:* • Banana	*Vegetables:* • Cucumber • Zucchini • Potato
Grass	*Pitted fruits:* • Cherry • Peach	*Other fruit:* • Kiwi • Orange • Tomato • Watermelon	*Vegetables:* • Potato
Mugwort	*Vegetables:* • Bell pepper • Broccoli • Cabbage • Cauliflower	• Chard • Garlic • Onion • Parsley	*Spices:* • Aniseed • Caraway • Coriander • Fennel • Black pepper

^*^Mouth or throat itching from peanut, soybean and nuts may also be an initial manifestation of a more serious food allergy with the potential for anaphylaxis. Refer the patient to an allergist/immunologist if such symptoms are noted

### Diagnosis

The diagnosis of food allergy requires a detailed history and physical examination, as well as diagnostic tests such as a skin prick test (SPT) and/or food-specific serum IgE assessment. In some cases, an oral food challenge (OFC) may also be required [17, 18].

#### History

The diagnosis of an IgE-mediated food allergy can sometimes be ruled out on history, based on symptoms or timing. For example, “mental fog” and headache are not compatible with an IgE-mediated allergic reaction and do not warrant allergy testing. It is important to inquire about all suspected culprit foods and discuss the manner of food preparation (e.g., cooked, raw, added spices or other ingredients). While pre-test probability is statistically higher for allergens with high known prevalence (Table 1), it is possible to become allergic to any food protein. However, pre-test probability of allergy will be lower for foods with high acid or enzyme content, such as tomatoes, strawberry, raw eggplant, or pineapple, which can induce direct mast cell degranulation. These are not generally associated with anaphylaxis (see *Anaphylaxis* article in this supplement).

Clinicians should inquire about the nature of symptoms (Fig. 1), the time of onset of symptoms in relation to food exposure, symptom duration, and symptom severity, as well as the reproducibility of symptoms in the case of recurrent exposures. Except in the case of alpha-gal, or co-factor dependent food allergies, symptoms that begin more than 2 h after the ingestion of a food are generally not attributed to an IgE-mediated allergy.

Having tolerated the food prior to the reaction does not rule out the suspected food as a potential culprit, since allergic sensitization could have developed since that previous exposure. However, as a general rule, a food that has been eaten in the same amount and tolerated since the reaction occurred can be ruled out as a culprit, unless the reaction was dependent on a co-factor that was not present on the subsequent ingestion.

#### Skin prick test (SPT)

The SPT is a rapid, safe, and sensitive method for measuring the presence of food-specific IgE on patient mast cells [31]. It consists of applying the specific allergen to the skin, and pricking the skin superficially to allow contact between mast cells and the allergen. A positive SPT appears as a wheal (measuring 3 mm or greater than the negative control) and flare reaction. A positive SPT only indicates the presence of food-specific IgE on mast cells and does not necessarily mean that there is a food allergy. It must be interpreted in the context of history, and possibly other diagnostic tests such as OFC. SPT has a sensitivity of approximately 90% for food allergy; however, its specificity is only around 50%, although specificity improves with a larger wheal size [31].

As a general rule, allergy testing serves to confirm a compatible history and is not sufficient alone for diagnosing food allergy [17, 32]. To minimize false positive results, clinicians should only conduct SPT for those foods that are implicated by the clinical history. On the other hand, a negative SPT generally confirms the absence of IgE-mediated reactions [17, 32]. An exception is PFAS, in which skin tests are usually negative to commercial food extracts but are positive to the fresh or frozen food [33]. It is also important to note that certain medications, such as antihistamines and tricyclic antidepressants, can interfere with SPTs, and these medications should be discontinued for a few days (or more) before testing [34, 35]. Prior discussion with the treating physician may be necessary before discontinuing certain medications, or diagnostic testing not affected by medications may be considered.

#### Serum-specific IgE

As an alternative or in conjunction with SPT, clinicians can also measure the levels of food-specific IgE in serum [17, 31]. Serum-specific IgE testing is readily available and is not dependent on the technician’s ability and the assessor’s interpretation. However, it can become expensive when multiple allergens are tested simultaneously. As with SPT, higher levels of specific IgE are associated with greater positive predictive values. Serum-specific IgE tests are useful for following an allergy to help determine whether it has been outgrown or to assess patient suitability for OFC or oral immunotherapy (OIT) (see *Oral Immunotherapy* article in this supplement). One limitation is that they have lower sensitivity in patients with low total IgE, which can lead to false negatives. Conversely, very high total IgE will decrease test specificity. This is particularly problematic in patients with atopic dermatitis (AD) (see *Atopic Dermatitis *article in this supplement), who may develop low-level sensitization to many foods, yet not be allergic to these foods. Panel testing of multiple foods in AD is not recommended as it is likely to lead to false food allergy labels and unnecessary food avoidance, putting the patient at increased risk of actually developing a food allergy [36, 37].

Component resolved diagnostic testing (CRD) consists of measuring the serum-specific IgE to each protein separately in a given food [38]. Molecular allergens available for CRD for common IgE-mediated food allergies are shown in Table 3 [39, 40]. CRD helps differentiate between allergy to heat-labile vs. heat-stable proteins. For example, egg-allergic patients who are allergic to ovalbumin but not to ovomucoid will generally react to fresh eggs but tolerate them in baked goods. CRD can also identify cross-reactive specific components to similar allergens from different pollen species or foods. For peanut, specific IgE against Ara h 8 protein in a patient allergic to birch is suggestive of PFAS, whereas specific IgE against Ara h 2 is the most consistent marker for predicting anaphylactic peanut allergy, as opposed to PFAS. As with specific IgE to whole peanut, the specificity of specific IgE against Ara h 2 increases with higher values and should always be interpreted in light of the clinical presentation [38].

**Table 3:** Molecular allergens available for CRD for common IgE-mediated food allergies [39, 40]

Allergic component	Protein family or function	Features
**Peanut**		
Ara h 1	SSP, 7S globulin (stable protein)	• Resistant to heat and enzymes • Sensitization associated with systemic reactions
Ara h 2	SSP, 2S albumin (stable protein)	
Ara h 3	SSP, 7S globulin (stable protein)	
Ara h 6	SSP, 2S albumin (stable protein)	
Ara h 8	PR-10 (labile protein)	• Cross reactive with birch pollen allergen Bet v1-homologue • Predominantly unstable and associated with less severe reactions such as PFAS
Ara h 9	LTP (stable protein)	• Increased stability to heat and proteases • At risk of systemic reactions, and co-factor-dependent anaphylaxis
**Hazelnut**		
Cor a 1	PR-10 (labile protein)	• Often associated with clinical tolerance or mild oral symptoms suggestive of PFAS
Cor a 8	LTP (stable protein)	• Associated with both mild and severe reactions, and co-factor-dependent anaphylaxis
Cor a 9	SSP (stable protein)	• Associated with increased risk of severe reactions
Cor a 14	SSP (stable protein)	
**Cow’s milk**		
Bos d 4	Alpha-lactalbumin	• ~ 65% of whey,* present in the milk of almost all mammals • Sensitization to this protein is generally associated with being able to tolerate milk in baked (but not fresh) form
Bos d 5	Beta-lactoglobulin	• ~ 25% of whey,* not present in human breast milk • Sensitization to this protein is generally associated with being able to tolerate milk in baked (but not fresh) form
Bos d 6	Bovine serum albumin	• ~ 8% of whey,* is one of the major beef allergens, responsible for cross-reactivity between CM and raw beef
Bos d 8	Casein	• Resistant to high temperatures • High sequence homology (> 85%) with protein from goat and sheep • Associated with higher risk of reaction to all forms of milk
**Hen Eggs**		
Gal d 1	Ovomucoid	• White-serine protease inhibition activity with high resistance to heating and chemical denaturation • Highly allergenic, correlated to high risk for reaction to all forms of egg
Gal d 2	Ovalbumin	• Serum protease inhibitor, heat-labile • Most abundant egg white protein • Correlated with risk for clinical reaction to raw or slightly heated egg and certain vaccines
Gal d 3	Conalbumin	• Low resistance to heating and chemical denaturation
**Soybean**		
Gly m 4	PR-10	• Cross-reactive allergen • Reactions in Birch allergic patients
Gly m 5	SSP, beta conglycinin (7S globulin)	• Major allergens • Implicated in primary sensitization • High stability to both heat exposure and gastric digestion • Associated with severe reactions
Gly m 6	SSP, glycinin (11S globulin)	
**Wheat**		
Tri a 14	Non-specific LTP	• Relevant food allergen in Italian wheat allergic patients • Also associated with baker’s asthma (rhinitis or asthma symptoms caused by inhaling wheat particles)
Tri a 19	Omega-5 gliadin	• Major allergen in wheat-dependent exercise-induced anaphylaxis • Relevant allergen in young children with immediate allergic reactions to ingested wheat
nTri aA TI	Alpha-amylase inhibitor protein	• Involved in both food allergy and wheat-dependent exercise-induced anaphylaxis

^*^Whey protein is susceptible to heat and loses ability to bind to IgE antibodies after 15–20 min of boiling at > 90 °C

LTP: lipid transfer protein; PFAS: pollen-food-allergy syndrome; PR-10: pathogenesis-related class 10 protein belonging to the Bet v1 homologous family; SSP: seed storage protein

#### Oral food challenge (OFC)

If there is clinical suspicion of food allergy but the diagnosis is uncertain based on SPT and/or specific IgE results, an OFC may be appropriate. In this procedure, the patient eats the suspected food in incrementally increasing doses, up to a serving size, under close medical supervision. If symptoms occur, they discontinue the challenge and treat the reaction. In some settings, including research, OFCs are often performed in a double-blinded manner, which is the gold standard for food allergy diagnosis.

OFCs should only be conducted by healthcare professionals who are experienced with food allergy and anaphylaxis management and have established procedures for conducting these challenges [41]. In addition, OFCs must be conducted in an office- or hospital-based setting with resuscitation equipment. Documentation of informed consent prior to the challenge should detail that the risks and benefits of the procedure were explained to the patient or caregiver, and that these risks were understood. Healthcare professionals conducting OFCs should also have an established plan for advising the patient based on the outcome of the challenge.

#### Reactivity threshold testing

There is increasing interest in using OFCs to document patients’ reactivity thresholds to help guide and individualize risk management strategies. An important caveat to threshold testing is that these can fluctuate over time, especially in the presence of co-factors like illness and exercise, and there is no consensus on the ideal “buffer zone” below which the patient could introduce the food. This said, approximately half of patients with IgE-mediated food allergies can consume a sizeable, non-trace portion of their allergen without reacting [42]. Practice is evolving to allow more flexibility and permissive approaches regarding trace exposure tailored to individual thresholds, preferences and tolerance to risk [43]. The caveat is that lack of symptoms with accidental exposure to small amounts should not be interpreted as the food allergy being “outgrown” as the patient may still react to higher doses.

### Management

**Fig. 2:**
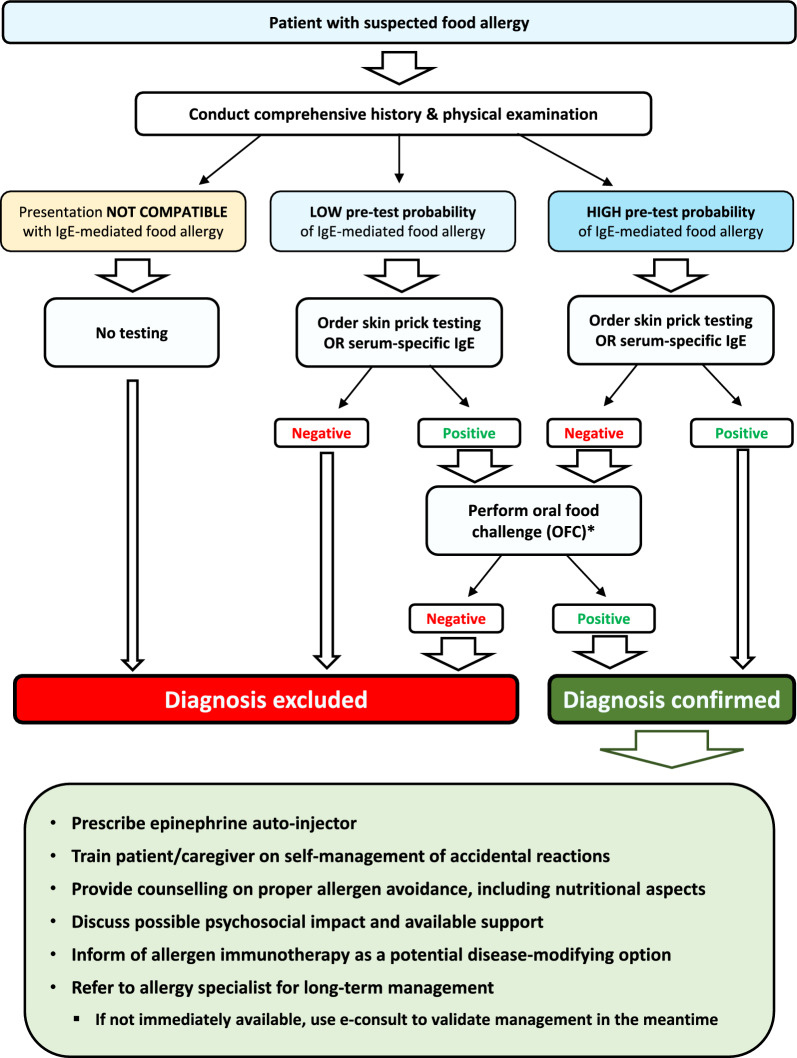
Simplified algorithm for the diagnosis and management of IgE-mediated food allergy. *OFCs should be performed by a trained healthcare professional in an environment that is adequate to treat anaphylaxis. IgE: immunoglobulin E; OFC: oral food challenge

A simplified algorithm for the diagnosis and management of food allergy is provided in Fig. 2.

#### Management of acute reactions

Clinicians should train all people with food allergy on the recognition of anaphylaxis and the proper self-administration of epinephrine (see *Anaphylaxis* article in this supplement). Intramuscular epinephrine into the lateral thigh is the treatment of choice for anaphylaxis [17, 18, 44, 45]. There are currently three epinephrine auto-injector (EAI) devices approved in Canada, in three dosages (0.15 mg and 0.30 mg for EpiPen and Allerject, and 0.30 mg and 0.50 mg for Emerade), that should be prescribed according to weight and availability [46–48] (note that at the time of publication of this manuscript, Emerade has been recalled and Allerject is difficult to access in Canada). According to the Canadian Society of Allergy & Clinical Immunology (CSACI), the 0.15-mg dosage should be used in children weighing < 25 kg and the 0.3-mg dose should be used in patients weighing ≥ 25 kg [45]. When available, the 0.50-mg dosage should be used in individuals weighing > 45 kg. These weight thresholds are lower than those indicated by the EAI manufacturers. In patients weighing < 7.5 kg, use of the 0.15-mg EAI is generally preferable to having parents use a syringe with the prescribed dose, due to the high risk of error with the latter [49].

Clinicians should advise patients not to store EAI in spaces subject to extreme cold or heat. They should also remind patients to replace EAI before they expire or if the solution changes color or goes cloudy.

Many patients hesitate to administer epinephrine for anaphylaxis because they worry about the potential side effects or believe its use necessitates a follow-up visit to the emergency department. However, in many cases, intramuscular epinephrine is very safe and does not require subsequent medical observation or cardiac monitoring. Thus, the latest recommendations from the CSACI are to administer epinephrine without delay whenever there is evidence of anaphylaxis [50]. At-home management, without emergency medical service (EMS) activation, is appropriate in the following circumstances [50]:

- Patient/caregiver comfort level with the recognition and management of anaphylaxis, in particular the prompt and correct use of EAI
- Immediate access to at least two, in date, weight appropriate doses of EAI
- Absence of risk factors for a biphasic reaction (i.e., a prior biphasic reaction, a moderate-to-severe reaction, delayed use of epinephrine [>60 min] or requirement of more than one dose of epinephrine)
- Absence of risk factors for severe anaphylaxis outcomes (i.e., cardiovascular disease, asthma [especially active or poorlycontrolled], mastocytosis)
- Symptom resolution with one dose of epinephrine administration
- Patient/caregiver preference

In all other circumstances, EMS should be activated and patients should be transported to hospital for evaluation and observation. Patients may then be observed at home, provided they experience a rapid and durable response to treatment.

The clinician should work with the patient and caregivers to prepare a concise, written anaphylaxis emergency plan for the treatment of allergic reactions resulting from accidental exposure to the food, with copies made available to appropriate community members (e.g., caregivers, daycare providers, teachers, employers). Examples of anaphylaxis emergency plans are available for download in both English and French from Food Allergy Canada [51].

#### Long-term management

According to the CSACI: “The ultimate goal of food allergy care should be the empowerment of patients and their caregivers to manage the risk of food-allergic reactions, reduce food-related anxiety and achieve a sense of control over their condition. This can be achieved in different ways for different patients” [52].

The two main long-term management options for patients with food allergy are food allergen avoidance and OIT (please refer to *Oral Immunotherapy* article in this supplement). Tactful and empathic shared decision-making with patients, their caregivers, and the OIT provider is necessary [52]. A review of pros and cons should be contextualized within the patient’s specific situation and should take into account their personal goals and preferences. Clinicians and parents/caregivers should be aware that the disease-modifying effect of OIT is conditional on its early start in infancy, and that the window for inducing remission closes as the child gets older.

While most patients with confirmed or suspected IgE-mediated food allergy will benefit from a consultation with an allergy specialist, this may not be feasible in areas with limited access. Building partnerships with allergists who offer remote support to primary-care providers and pediatricians is key to ensuring the best quality allergy care for patients in these areas.

A properly managed, well-balanced diet that excludes known allergens will help keep an individual free of food allergy symptoms while maintaining their nutritional status. In infants ≤ 1 year of age with cow’s milk allergy who need a breastmilk alternative, guidelines recommend hypoallergenic extensively hydrolyzed formulas or amino-acid based formulas [53]. Partially hydrolyzed CM formulas, mammalian milk, or for infants under 6 months, soy-based formulas are not recommended in these infants due to the potential for allergic reactions [53].

Patients and caregivers require education on how to effectively avoid food allergens. Registered dietitians with specialised food allergy training and expert-informed peer-support groups can be very useful in helping them learn how to read food labels, avoid cross-contamination, manage the risk of trace exposure, and effectively communicate and request accommodation for their food allergy needs, including in community settings such as childcare, schools, and workplaces [54, 55].

Food allergy is associated with a significant psychosocial burden for patients and families, including food allergy anxiety regarding exposures outside the home, the risk of anaphylaxis as well as the social limitations of the condition [56, 57]. Mental health professionals and expert-informed peer-support programs may play an important role in helping patients and families manage the psychosocial effects of food allergy [58]. Following life-threatening reactions, clinicians should not only re-assess the context in which these occurred, but also evaluate the patients and caregivers for signs and symptoms of severe anxiety and post-traumatic stress disorder, and refer to a mental health professional for support when indicated [59].

Once evidence exists that the food allergy has resolved, the relevant food should be reintroduced [17, 18]. To this end, clinicians should reassess patients with food allergy periodically (e.g., every 6 months in infants or toddlers, yearly in children and adolescents, and every 2–5 years in adults).

#### Biologics

There are several reports of biologics used either as monotherapy or as adjuncts to OIT to treat IgE-mediated food allergy [52, 53, 60]. Omalizumab – an anti-IgE monoclonal antibody that was first approved to treat allergic asthma – has been shown to significantly increase reactivity thresholds to food allergens in a dose-dependent manner [52, 53, 60]. The Food and Drug Administration (FDA) in the United States has recently approved omalizumab for the treatment of IgE-mediated food allergy in both adults and children aged 1 year or older [61]. Omalizumab is not yet approved in Canada for this indication.

Clinical trials are currently underway to investigate the potential efficacy of short courses of other biologics targeting the cellular immune response, such as dupilumab, etokimab, and abatacept, to improve immunomodulatory and sustained tolerance outcomes of OIT.

### Prognosis

The prognosis of food allergy is complex and dependent on the particular food. CM and egg allergy typically present in the first year of life. Although some children may outgrow these allergies by early school age, others may not develop tolerance until their teenage years or may remain allergic for life. For CM, studies have reported that 19% of subjects achieve tolerance by age 4 years, 42% by age 8 years, 64% by age 12 years, and 79% by age 16 years [62]. For egg allergy, 4% achieve tolerance by age 4 years, 12% by age 6 years, 37% by age 10 years, and 68% by age 16 years [63]. In contrast, allergy to peanut, tree nuts, fish, and shellfish are generally lifelong, although 20% of individuals may outgrow peanut allergy [64]. Peanut and tree nuts are responsible for the most serious allergic reactions and food allergy-related fatalities [65, 66].

### Prevention

Given the high burden associated with food allergy, primary prevention has become an important public health goal. Current evidence suggests that both early introduction and regular ingestion of allergenic foods (once introduced) are imperative for the primary prevention of food allergy [67, 68]. For more detailed information on prevention, please see the article entitled, *Primary Prevention of Food Allergy: Beyond Early Introduction,* in this supplement.

## Conclusions

IgE-mediated food allergy is an important clinical problem of increasing prevalence. Diagnosis primarily relies on a careful history, confirmation with diagnostic tests, such as SPT, food-specific serum IgE assessment, and if indicated, OFCs. All patients must be trained on the proper avoidance of the culprit food(s) and timely administration of epinephrine for anaphylaxis. OIT is an option for inducing desensitization and promotes clinical remission in infants and toddlers. Further insights into the pathophysiology of food allergy and anaphylaxis will lead to the development of improved methods for prevention, diagnosis, and management.

## Data Availability

Not applicable.

## Abbreviations

AD: Atopic dermatitis
CM: Cow’s milk
CSACI: Canadian Society of Allergy & Immunology
EAI: Epinephrine auto-injector
EMS: Emergency medical services
GA2LEN: Global Allergy and Asthma European Network
GI: Gastrointestinal
IgE: Immunoglobulin E
IgG: Immunoglobulin G
SPT: Skin prick tests
OFC: Oral food challenge
OIT: Oral immunotherapy
PFAS: Pollen-food allergy syndrome
