# Publisher Correction: Immunoglobulin E (IgE)-mediated food allergy

**DOI:** 10.1186/s13223-025-00974-3

**Published:** 2025-06-25

**Authors:** Philippe Bégin, Susan Waserman, Jennifer L. P. Protudjer, Samira Jeimy, Wade Watson

**Affiliations:** 1 Division of Clinical Immunology and Allergy, Department of Medicine, Université de Montréal, Montréal, Québec Canada; 2 Division of Clinical Immunology and Allergy, Department of Medicine, McMaster University, Hamilton, ON Canada; 3 Department of Pediatrics and Child Health, College of Medicine, Rady Faculty of Health Sciences, University of Manitoba, Winnipeg, MB Canada; 4 Children’s Hospital Research Institute of Manitoba, Winnipeg, MB Canada; 5 Division of Clinical Immunology and Allergy, Department of Medicine, Western University, London, ON Canada; 6 Division of Allergy, Department of Pediatrics, Dalhousie University, IWK Health Centre, Halifax, NS Canada

**Publisher Correction: Allergy, Asthma & Clinical Immunology (2024) 20:75**

10.1186/s13223-024-00930-7

Following publication of the original supplement article [1] we became aware that the below bullet points were inadvertently omitted from the third paragraph of the “Management of acute reactions” section. These should appear after the sentence: 

At-home management, without emergency medical service (EMS) activation, is appropriate in the following circumstances [50]:

- Patient/caregiver comfort level with the recognition and management of anaphylaxis, in particular the prompt and correct use of EAI;
- Immediate access to at least two, in date, weight-appropriate doses of EAI;
- Absence of risk factors for a biphasic reaction (i.e., a prior biphasic reaction, a moderate-to- severe reaction, delayed use ofepinephrine [> 60 min] or requirement of more than one dose of epinephrine);
- Absence of risk factors for severe anaphylaxis outcomes (i.e., cardiovascular disease, asthma [especially active or poorlycontrolled], mastocytosis);
- Symptom resolution with one dose of epinephrine administration;
- Patient/caregiver preference.

The original article has been corrected.
